# Supplementary material for: Shift in Potential Malaria Transmission Areas in India, Using the Fuzzy-Based Climate Suitability Malaria Transmission (FCSMT) Model under Changing Climatic Conditions
Source: Int J Environ Res Public Health. 2019 Sep 18;16(18):3474. doi: 10.3390/ijerph16183474 (PMC6766004; doi:10.3390/ijerph16183474)
Supplement: Supplementary file 1 [file ijerph-16-03474-s001.pdf]

International Journal of
Environmental Research and Public Health

Supplementary

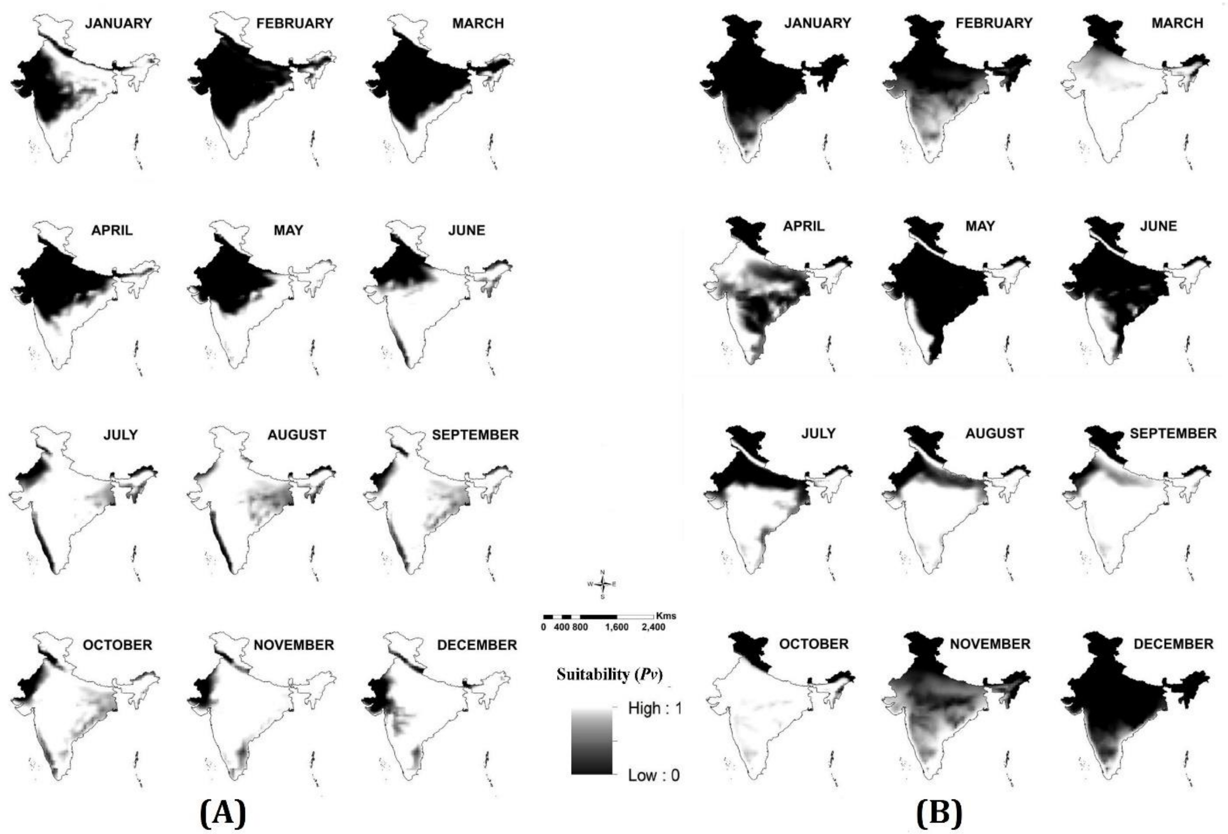

Figure S1. Temperature and RH suitability for baseline years and projected 2030s: Fuzzy partitioning of Temperature and RH distribution with sinusoidal membership function generate spatially smooth suitability maps for Pv and Pf malaria transmission scenarios for both time periods. Areas, where temperature/RH are most suitable (fuzzy value of 1) in a month, are denoted with lighter tone that gradually increase to darker gray tone indicating locations where transmission suitability for malaria is least (fuzzy value of 0). Temperature and RH suitability for malaria transmission across the country vary between months. Between June to December, more than 70% of the country has suitable RH, while the month of March and July to October are having suitable temperature. One such scenario is given here, where maps represent 2030s monthly suitability maps for *Pv* malaria transmission (A) based on RH and (B) Temperature.

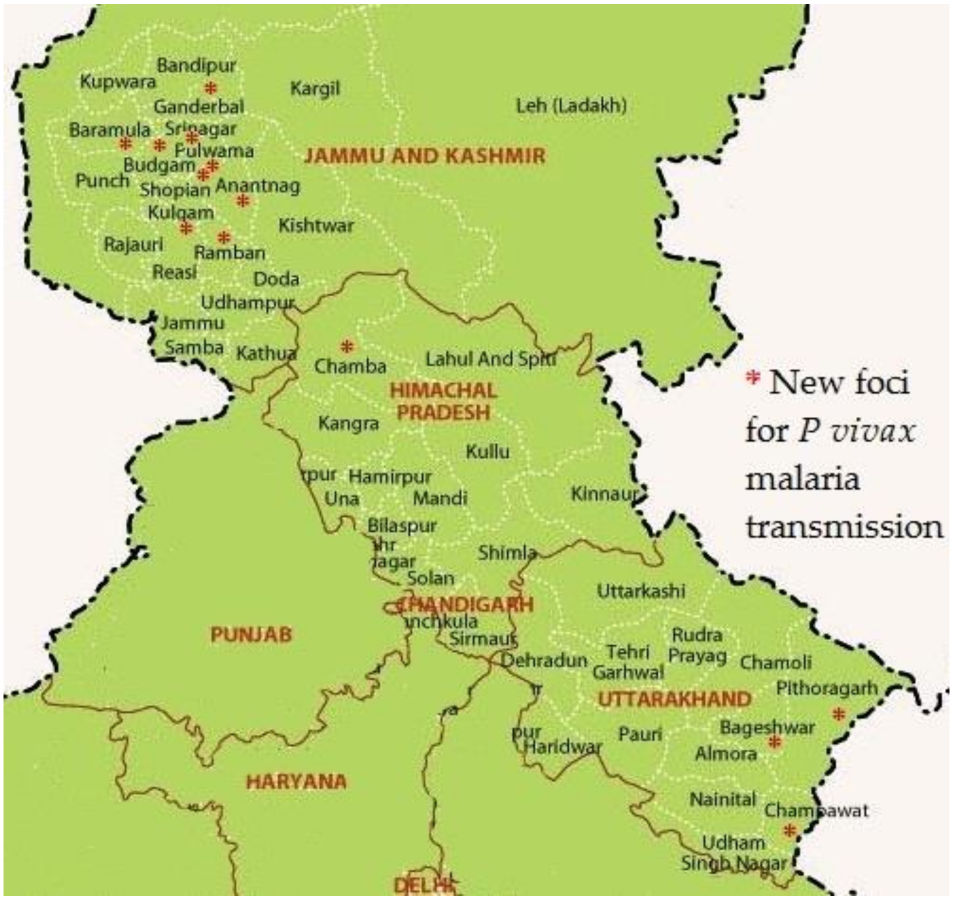

Figure S2. Map showing the potential districts as new foci for Pv malaria transmission. New foci are located in three states of India, namely, Jammu and Kashmir, Himachal Pradesh and Uttarakhand.
